# Late Recovery from Stuttering: The Role of Hand Dominancy, Fine Motor and ‎Inhibition Control

**Published:** 2016-01

**Authors:** Hiwa Mohammadi, Habibolah Khazaie, Mansour Rezaei, Mohammad Taghi Joghataei

**Affiliations:** 1 Department of ‎Neuroscience, School of ‎Advanced Technology in ‎Medicine, Iran University ‎of Medical Sciences, ‎Tehran, Iran; 2 Sleep Disorders Research Center, Kermanshah University of Medical Sciences, Kermanshah, ‎Iran; 3 Department of ‎Biostatistics, School of ‎Medicine, Kermanshah ‎University of Medical ‎Sciences, Kermanshah, ‎Iran

**Keywords:** Hand Fine Motor Control, Inhibition Control, Recovery from Stuttering

## Abstract

**Objective: **There are controversial reports about factors that affect recovery from stuttering. In the ‎present study, the effect of hand dominancy, fine motor and inhibition control on late ‎recovery from stuttering was investigated among a group of Kurdish-Persian children who ‎stuttered in Iran.‎

**Method:** Twenty-two Kurdish-Persian children aged 7-14 years who stuttered were followed for 6 ‎years. Based on the evaluation of three experienced speech therapists and parental judgments, ‎these children were classified into recovered or persistent groups. Data about fine motor ‎control of hand and inhibition control were obtained, using Purdue Pegboard and Victoria ‎Strop Color Word Tests, respectively. Risk factors including sex, age, and family history of ‎stuttering, handedness, inhibitory control and fine motor control of hand were compared ‎between the groups and modeled to predict recovery from stuttering using logistic regression.‎

**Results:** From the 22 participants, 5 (22.7%) recovered from stuttering. The recovered and persistent ‎groups did not show significant differences in the interference effect. By dividing the scores ‎of the Purdue Pegboard tests to the right and left hand, we created a new Handedness Index ‎‎(HI). HI was significantly higher in the recovered group. The score of right hand was higher ‎than the left in the recovered group, but no difference was found between the two hands in ‎the persistent group. Among the investigated risk factors, only HI could predict the recovery ‎from or persistency of stuttering with 94% sensitivity and 84% specificity.‎

**Conclusion**: Handedness Index can predict the recovery from stuttering significantly among children who ‎stutter.‎

Stuttering is a dynamic speech motor disorder that involuntarily interrupts the ‎temporal aspects and coordination between the subsystems of speech structures (1, 2). The ‎disorder affects 1% of the adult population with an estimated incidence of 5-8% (3, 4). Many ‎studies have investigated the different aspects of stuttering, but the causes of this disorder are ‎still unknown. Nevertheless, a decrease with age was established in the prevalence of ‎stuttering. Previous studies have reported high levels of spontaneous recovery from stuttering ‎‎3 to 5 years after onset (3). However, the biological and environmental factors determining ‎the persistency of or recovery from stuttering are not yet well-understood (3). Although it has ‎been reported that most natural recovery takes place before the age of seven, recovery can ‎happen at any age (5). According to the time elapsed from the onset of stuttering, two types ‎of recovery, early and late, have been identified (6). Very few studies have investigated late ‎recovery from stuttering (7). ‎

Several factors such as sex, age of onset, family history of the disorder and severity of ‎stuttering have been reported as predictors for persistency of or recovery from stuttering (3, ‎‎^8^). Some studies reported the relation between inhibitory control and dysfluency in normal ‎children and those with attention deficit hyperactivity disorder (9). It has been reported that ‎self-regulation and inhibition problems as well as high nonfluency may have similar ‎pathogenic mechanisms (10). Based on these suggestions, a few recent studies reported lower ‎inhibitory control in people who stutter compared to those who do not (11). However, the ‎effect of inhibitory control on recovery from stuttering has not been studied yet. ‎

Another important factor is handedness and hand motor control. Studies have shown ‎that left handedness is more prevalent in people who stutter, and it has been suggested that ‎right handedness increases the chance of recovery (12). Therefore, an etiological relation ‎between complication of cerebral dominance and stuttering such as disruption, abnormality, ‎or abnormal pattern of brain laterality has been proposed. Also, neuroimaging studies have ‎indicated hyper-activation of the right hemisphere and bilateral cerebellar activity in people ‎who stutter (13-15). Moreover, motor disruptions in speech and non-speech orofacial and ‎finger movements have been reported (16). ‎

Bilingualism, as a multidimensional and complicated phenomenon, can also affect the ‎onset and development of stuttering (17, 18). Earlier studies have reported a high prevalence ‎of stuttering among bilinguals compared with monolinguals (19, 20), although this has not ‎been confirmed in subsequent studies (21). Regardless of the controversies about the role of ‎bilingualism on the development of stuttering, few studies have investigated recovery from ‎stuttering in bilinguals. Howell, Davis, and Williams (2009) reported an increased risk of ‎stuttering and a lower chance of recovery from stuttering among bilinguals compared to ‎monolinguals (22). They suggested further studies on recovery from stuttering among ‎bilinguals. ‎

Considering the above mentioned, we aimed to investigate the late recovery from ‎stuttering in a group of Kurdish-Persian bilingual children who stutter. Moreover, in addition ‎to previously investigated factors such as age, sex, and family history of the disorder, we ‎investigated the role of inhibition control, fine motor skills, and handedness on recovery from ‎stuttering. ‎

## Materials and Method

***Participants:***

Thirty-seven Kurdish-Persian bilingual children (26 boys and 11 girls) aged 7-14 years ‎who suffered from stuttering were enrolled in the study during 2007. The study was ‎conducted in Javanroud, located in Kermanshah province, West Iran. The first language of ‎the city is Kurdish and children usually learn Persian as their second language at school. All ‎of our participants were born to Kurdish native parents. They were Kurdish native speakers ‎and had learned Persian from television, media, and formal education at school. Teachers had ‎referred them as people with stuttering in 2007. Then they were invited to participate in the ‎study. After obtaining the written informed consent from all parents, spontaneous speech ‎samples in both languages were videotaped. Kurdish and Persian speech samples were ‎obtained by Kurdish and Persian interviewers, respectively, under friendly interviewing ‎conditions. Speech samples were obtained using methods such as story telling using serial ‎pictures and free discussions about interesting topics for students. ‎

To diagnose people who stutter, three registered speech language pathologists who had ‎at least five years of experience working with Kurdish-Persian bilinguals who stutter analyzed ‎four spontaneous speech samples (two samples in each language). One of the speech language ‎pathologists was a Kurd and the other two were Persian native speakers. Finally, participants ‎who were identified as stutterers by teachers, diagnosed as stutterers in both languages by the ‎three speech language pathologists based on the evaluation of the four speech samples in ‎Kurdish and Persian, and confirmed as stutters by parents continued their participation in the ‎study. According to this multistep procedure, 37 students were identified as stutterers, but ‎two were excluded from the study. One of the boys was not identified as a stutterer by his ‎parents and the other had only paused before speaking Persian words but did not show any ‎signs of dysfluency in Kurdish.‎

***Procedure:***

The parents of the 35 participants completed questionnaires consisting of information ‎about demographic characteristics and stuttering history. The family history of stuttering was ‎also obtained from the parents using a checklist that investigated first and second degree ‎relatives with stuttering. Parents were asked to write any relatives that had stuttering or ‎recovered from it in the checklist. ‎

Six years later in 2013, the 35 children who stuttered were contacted and invited to ‎participate in the second phase of the study. Since the researchers could not contact and find ‎the 13 families, ultimately, the data of 22 participants including 14 boys and 8 girls were ‎collected. The participants were examined again in the four speech samples in both languages ‎by previous clinicians and methods. Participants who were identified as non-stutterers in both ‎languages by the three speech therapists and were confirmed to be fluent speakers by their ‎parents were categorized as the recovered group. Therefore, the participants were divided into ‎two recovered and persistent groups. Through a careful interview, data were obtained on ‎medication and speech therapy from both groups. Then, handedness was assessed by the ‎Persian version of The Edinburgh Handedness Inventory. This inventory includes questions ‎about which hand is used by the subject for several everyday activities. Each question is ‎scored on a five-point Likert scale from always right (+10) to always left (−10). After the ‎summation of scores, +40 and -40 were considered as cutting points for truly right and truly ‎left laterality, respectively. The scores between these two points were considered as ‎ambidextrous (23).‎

Later, having used the Lafayette Instrument Purdue Pegboard Test Model 32020, we ‎evaluated the finger/hand function, dexterity, and laterality in the participants. The test has ‎been used widely to evaluate hand laterality and motor control in a broad range of brain ‎damages and dysfunctions (24-26). The subjects completed three separate test batteries ‎including right hand (30 seconds), left hand (30 seconds), and assembly (60 seconds) tests ‎according to the instructions. By dividing the right to left hand scores, we created a ‎handedness index. Finally, the inhibition control among the participants was investigated ‎using the computer version of the Victoria Stroop Color Word Test (27). The test was used to ‎evaluate the executive function, cognitive flexibility, inhibition ability, and attention deficits ‎in many neurological disorders (28, 29). The numbers of correct answers, errors, reaction time ‎and interference are the criteria for scoring. Recently, the test was used and standardized ‎among Iranian bilingual population (30). ‎

The software version of Persian Victoria Sroop Color Word test (31) and a lap top ‎computer with 14" LCD monitor were used. In the first stage that lasted 45 seconds ‎participants were asked to choose the color of the 16 circle shown on the screen in blue, red, ‎yellow and green. Answers could be selected by V, B, N, M keys on the keyboard covered by ‎blue, red, yellow and green, respectively. In this first stage, we aimed to test and practice the ‎color perception and place of keys. For testing the participants’ understanding of the purpose ‎of test, the first stage was followed by another preliminary trial that lasted 45 seconds. Eight ‎congruent and eight incongruent color names were presented on screen and the participant’s ‎had to identify the color and not the meaning of the words. The scores of this stage were not ‎involved in the analysis. In the next step, 48 congruent and 48 incongruent chromatic words ‎were presented randomly. Each word was presented for two seconds on the screen with 0.8 ‎seconds intervals. In a real-time analysis manner, the software measures total time, mean ‎reaction times, and numbers of no response, correct, and incorrect (errors) answers for ‎congruent and incongruent color names, separately. ‎

Finally, all data were analyzed using SPSS software, Version 20. The recovery rate ‎according to sex, age, and the family history of stuttering was investigated using Chi-square ‎and Fisher's exact tests. The scores of the two groups in the Purdue Pegboard and Stroop ‎Word Color Tests were analyzed by independent and paired sample t, Mann-Whitney U and ‎Wilcoxon tests. A logistic regression model was employed to determine the factors that could ‎predict recovery from stuttering.‎

## Results

**Fig1:**
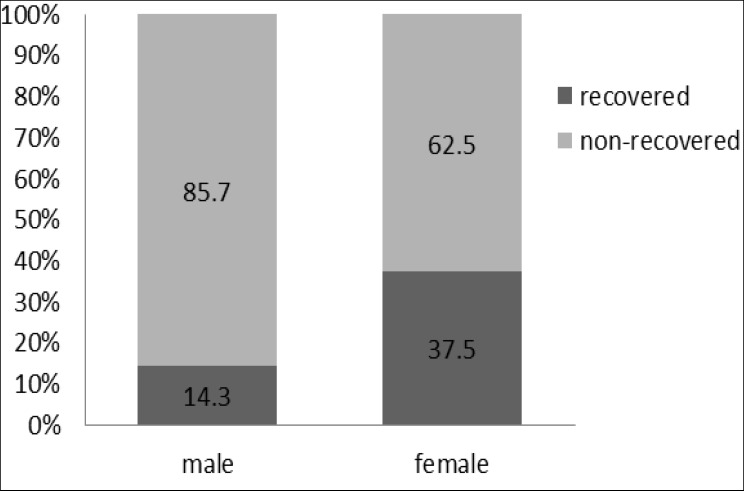
recovery from stuttering in male/female

Twenty-two children aged 7-14 years with a mean±SD age of 9.2±1.79 years were ‎followed from 2007 to 2013. According to the result of the Edinburgh Handedness ‎Inventory, only two participants in the persistent group were ambidextrous and all others in ‎both groups were right handed. After the-six-year follow-up, five (22.7%) of the 22 ‎participants had recovered from stuttering. However, the rate of recovery for the girls was ‎slightly higher than boys (p = 0.309; Figure 1). 

**Fig 2:**
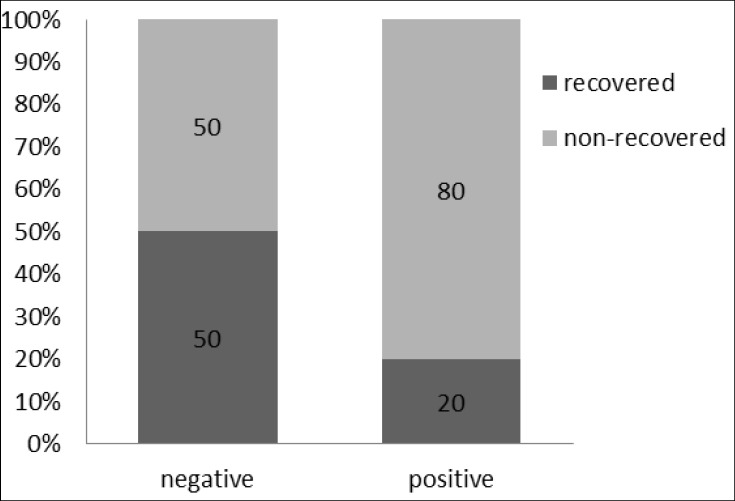
Late recovery from stuttering in subjects with negative / positive history of stuttering in their family

Four of the five recovered children and 16 of ‎the persistent participants reported a family history of stuttering (p = 0.41; Figure 2). Overall, ‎only two out of the 22 participants did not have a family history of stuttering. In order to ‎compare the age-related recovery rate, the participants were divided into two separate age ‎groups (7-10 and 11-14 year-olds). No significant difference was observed in the recovery ‎rate between the two age groups (p = 0.675, Fisher's exact test). ‎

‎ Among Purdue Pegboard subtests, only the scores of the left hand in the persistent ‎group was significantly higher than the recovered group (p = 0.00954) and the differences in ‎the other subtests were not significant. Because the difference in left hand scores between the ‎two groups might have been attributed to the two ambidextrous participants in the persistent ‎group, they were omitted from data, and comparisons were performed again. Significant ‎statistical differences were also found between the two groups after repeating the analysis (p ‎‎= 0.00933). On the other hand, the right hand scores of the recovered group were ‎significantly higher than their left hand scores (p = 0.032). However, the same result were not ‎obtained in the persistent group (p = 0.455). The two groups differed significantly in the ‎handedness index (p = 0.005; Table 1). ‎

Stroop effect (Interference Effect) scores between the recovered (=1) and persistent ‎‎(=1.24) groups did not differ significantly. The subtests of the congruent part of the Stroop ‎Color-Word Test did not differ significantly between the two groups. The same results were ‎also observed for the incongruent parts (Table 2). ‎

‎Nevertheless, when the function of each group in congruent and incongruent steps ‎was compared, significant differences were found between these two steps in the persistent ‎group, but not in the recovered group in all five subtests (Table 3). We found that the ‎function of the persistent group in congruent steps was significantly better than incongruent ‎steps (p<0.05; Table 3).

The predictor variables including sex, family history of stuttering, age, interference ‎effect, scores of assembly subtest of Purdue Pegboard and handedness index were entered ‎into a logistic regression using enter and then forward stepwise model. According to the ‎results of the enter model, none of the variables could predict the recovery/persistency from ‎stuttering significantly. However, a good fit was observed between observed and predicted ‎conditions. The model correctly classified 100% of the persistent or recovered cases and had ‎a good sensitivity and specificity. The -2 log-likelihood (-2LL) statistic was 0.000, Cox and ‎Snell R2 was 0.658 and Nagelkerke R2 was 1.00, all confirming a good fit of the model to the ‎data. Using the forward stepwise model, the handedness index was a significant predictor of ‎recovery from/persistency of stuttering (p = 0.022; Table 4). Handedness index could predict ‎the recovery from or persistency of stuttering with 94% sensitivity and 84% specificity. ‎Results from the logistic regression model indicated that a unit increase in handedness index ‎increased the chance of recovery more accurately.

**Table1:** Comparison of the Purdue Pegboard Subtests scores between the recovered from and persistent to stuttering groups

**Groups**		**Recovered**	**Persistent**	**P value**
**Purdue Pegboard** **Subtests**	Right hand	14.4±0.89	14.5±1.5	0.858
	Left hand	12.8±0.44	14.35±1.16	0.00954
	P value	0.016	0.455	
	Handedness Index	1.1256±0.0704	1.0125±0.0695	0.005
	Assembly	26.6±6	27.47±3.28	0.673

**Table2:** Comparison of Stroop Color Words Subtests between the recovered from and persistent to stuttering groups

**Stroop Test**	**subtests**	**Recovered**	**Persistent**	**P-value**
**Congruent**	Total time (s)	45.8 ± 3.96	46.82 ± 6.45	0.742
	errors	0.2 ± 0.44	0.35±0.78	0.685
	Non- respond	0	0.18±0.39	0.335
	Correct response	47.8±0.44	47.47±0.87	0.432
	Reaction time (ms)	968±81	983±33	0.811
**Incongruent**	Total time (s)	47.6 ± 5.72	49.18±6.63	0.637
	errors	0.4±0.54	0.94±1.14	0.324
	Non- respond	0.8±1.09	0.82±1.33	0.972
	Correct response	46.8±0.83	46.26±1.78	0.507
	Reaction time (ms)	982±98	1017±127	0.580

**Table 3:** Comparison of Stroop Subtests scores between Congruent and Incongruent parts in the recovered from and persistent to stuttering groups

**Groups**	**Subtests**	**Congruent**	**Incongruent**	**p-value**
Recovered	Total time (s)	45.8 ± 3.96	47.6 ± 5.72	0.105
	Errors	0.2 ± 0.44	0.4 ± 0.54	0.374
	Non- responded	0	0.8 ± 1.09	0.178
	Correct response	47.8 ± 0.44	46.8 ± 0.83	0.089
	Reaction time (ms)	968 ± 81	982 ± 98	0.161
Persistent	Total time(s)	46.82 ± 6.45	49.18 ± 6.63	0.000
	Errors	0.35 ± 0.78	0.94 ± 1.14	0.039
	Non-responded	0.18 ± 0.39	0.82 ± 1.33	0.031
	Correct response	47.47 ± 0.87	46.26 ± 1.78	0.007
	Reaction time (ms)	983 ± 33	1017 ± 127	0.003

**Table 4:** Logistic regression analysis of the relationship between predictive variables and late recovery from stuttering using Forward Stepwise Model

Variable	LL	-2LL	Sig of -2LL	OR	CI	P-value
Handedness Index (HI)	-12.658	11.020	0.001	4.038E11	48.519-3.360E21	0.022

## Discussion

In the present study, the rate of recovery from stuttering among Kurdish-Persian bilingual ‎children who stuttered was investigated during a six-year period. All the participants were ‎Kurdish-Persian bilinguals who were diagnosed as stutterers between the ages of 7 and 14. ‎Data showed that only two participants received speech therapy intervention for a short time, ‎but they were not diagnosed as recovered in the final evaluation. Therefore, the rate of ‎recovery in the present study was investigated with respect to spontaneous or without formal ‎intervention recovery. Five (22.7%) of the 22 children who stuttered had recovered. ‎

The recovery rate from stuttering in the present study was lower than other studies on ‎younger children. Yairi and Ambrose reported a 74% recovery among 2-5 year-old children ‎who stuttered (32). In a six-year follow-up study on 23 participants with parental history of ‎stuttering who were in the initial stage of the disorder and by using parent indication method, ‎Kloth and colleagues reported a 70% recovery rate (33). Some other studies that explored the ‎rate of recovery 2 to 5 years after the onset reported 68% to 80% recovery from stuttering (5, ‎‎^34^-^36^). This discrepancy is consistent with the view that greatest recovery occurs when ‎children are younger. Contrary to studies on younger children, in a 10-year follow-up of ‎children aged 7-9 years who stuttered through their teenage years, Fritzell (1976) (cited by ‎Yairi & Ambrose, 2013) reported a recovery rate of 47% (32). Howell et al. reported a ‎recovery rate of about 50% after four years follow-up of 76 eight-year-old children who ‎stuttered (7). All these studies were conducted on monolingual population; however, similar ‎to our study, Howell et al. reported a 25% recovery rate from stuttering among 8-10 year-old ‎bilinguals from birth after four years of follow-up. They found that the recovery rate of ‎bilingual children who stuttered was significantly lower than monolinguals and bilinguals that ‎learned English as a second language at school (22). Considering that they merged the ‎bilinguals that learned English as a second language at school and monolinguals because of ‎the low numbers in the former group, when these speakers were divided into persistent and ‎recovered cases, it was impossible to compare the recovery rate in bilinguals that learned ‎English as a second language at school and the bilingual children who stuttered in this study. ‎Nevertheless, as the only study that investigated the recovery from stuttering among ‎bilinguals, and based on the similarity of age range between our study and theirs, it is ‎probable that bilingualism has a negative effect on the chance of recovery. ‎

The chance of recovery was neither dependent on the family history of stuttering nor ‎on age and sex. These results are in agreement with several previous studies (7, 8). Studies, ‎which investigated younger participants, showed that persistent children who stutter had ‎more stuttering relatives in their families (37, 5). However, some researcher argued that the ‎family history of stuttering probably is not a risk factor for persistency in older people who ‎stutter (7, 38). Logistic regression models also indicated that hand fine motor control ‎‎(assembly subtest of Purdue Pegboard test) and interference effect could not significantly ‎predict recovery from stuttering. Among the risk factors investigated here, only handedness ‎index could predict the recovery from or persistency of stuttering with 94% sensitivity and ‎‎84% specificity. This finding reveals the importance of handedness and brain laterality in the ‎development of stuttering.

Findings of Purdue Pegboard test revealed an interesting picture. In contrast to the ‎recovered group, there was no significant difference between right and left hands for the ‎persistent group in the Purdue Pegboard test and they did not show any asymmetry in ‎performance between the two hands. Our findings confirm previous research suggesting that ‎people who stutter have problems in complete laterality. In contrast to previous studies that ‎reported subtle deficit in fine motor control of people who stutter (39, 40), we found no ‎significant difference between right hand and assembly subtest of Purdue Pegboard test and ‎the persistent group performed the test as skillfully as the recovered. Likewise, some previous ‎reports did not find any differences in speech movements between children who stutter and ‎the control group (41). In a recent study, no significant difference was found between the ‎children who stuttered and those who did not in terms of the acoustic patterns they produced ‎in the diadochokinesis tasks (42). Some other researchers reported different results and found ‎that people who stutter exhibited longer finger reaction time compared to normal subjects. It ‎has been suggested that some people who stutter may have difficulty in the consistent ‎execution of motor control strategies common to both speech and non-speech movements ‎‎ (43). Slower finger and vocal reaction time were also reported by other researchers (44). In ‎contrast to these reports, our recovered and persistent groups had similar performance in terms ‎of reaction time of both congruent and incongruent trials of the Stroop Color Word Test. ‎

With respect to the Stroop Color Word Test, although the recovered group ‎performed both congruent and incongruent trials better than the persistent group, there was ‎no significant difference between the two groups in all subtests of the two trials. While the ‎recovered group did the two trails of the test similarly, an interesting finding was the ‎significant difference between congruent and incongruent trials in the persistent group. The ‎result indicated that the persistent group obviously performed the incongruent trails slower ‎and less proficiently than the congruent trails. Here the interference effect was revealed as an ‎increase in reaction times, and the persistent group needed to complete the incongruent ‎compared to congruent trials (45). Slower reaction time in doing incongruent trails may reflect ‎the interference effect in the persistent group and indicate that they need more time to inhibit ‎habitual responses. However, we found no significant differences between recovered and ‎persistent groups in the interference effect, which could be attributed to the small sample size. ‎These findings indicate the probable role of inhibition control in the occurrence of stuttering. ‎It has been reported that inhibitory control is a necessary factor for successful task ‎performance and plays an important role in the self-regulation of emotional states (46) and ‎coordination and integration of mental processes (47). Previous investigations also showed ‎that children who stuttered had lower ability in inhibitory control when doing the GO/NoGo ‎task (11). And the last but not the least is the fact that most differences in fine motor skills ‎and inhibition control were reported in studies comparing people who stutter with a normal ‎control group. ‎

## Limitations

Our study had some limitations. We had no normal control group. Moreover, the small ‎sample size and its further decline due to inaccessibility should be taken into account in ‎interpreting the results. Future studies could be done on the role of fine motor control, brain ‎laterality and inhibitory control by elaborate longitudinal studies on a larger sample using ‎accurate neuropsychological tests. ‎

## Conclusion ‎

In children who stutter, the rate of recovery from stuttering for the bilinguals may be ‎lower than the monolinguals. The chance of recovery neither depended on the family history ‎of stuttering nor on the age and sex of the participants. In terms of handedness, hand motor ‎control and inhibition, no significant difference was found between recovered and persistent ‎groups except for left hand function that was higher in the persistent group. The handedness ‎index that was obtained from dividing the motor function of right to left hand, could predict ‎the recovery from stuttering significantly and accurately. The persistent group may have ‎problems in hand function asymmetry and inhibitory control.‎
